# Supplementary material for: Understanding children’s experiences of self-wetting in humanitarian contexts: An evaluation of the Story Book methodology
Source: PLOS Glob Public Health. 2023 May 15;3(5):e0001194. doi: 10.1371/journal.pgph.0001194 (PMC10184904; doi:10.1371/journal.pgph.0001194)
Supplement: S2 File — Table A. Story Book sessions held in Cox’s Bazar. Table B. Answers given* on how the Hero feels after self-wetting. Table C. Answers given* on reactions of others to the Hero after self-wetting. Table D. Answers given* when asked why the Hero wet the bed. Table E. Answers given* when asked how the Hero could stop wetting the bed. (DOCX) [file pgph.0001194.s002.docx]

# S2 File: Data on sessions held in Cox’s Bazar

**Table A. Story Book sessions held in Cox’s Bazar.**

| **Session reference** | **CB 1** | **CB 2** | **CB 3** | **CB 4** | **CB 5** | **CB 6** | **CB 7** | **CB 8** |
| --- | --- | --- | --- | --- | --- | --- | --- | --- |
| Location | Camp 8E | Camp 8E | Camp 8E | Camp 8E | Camp 7 | Camp 7 | Camp 7 | Camp 7 |
| Participant gender | Boys | Girls | Girls | Boys | Boys | Girls | Boys | Girls |
| Participant age in years | 5 to 7 | 8 to 11 | 5 to 7 | 8 to 11 | 8 to 11 | 8 to 11 | 5 to 7 | 5 to 7 |
| Number of participants | 7 | 7 | 6 | 6 | 6 | 6 | 6 | 6 |
| Duration in minutes | 131 | 115 | 50 | 71 | 155 | 135 | 131 | 141 |

**Table B. Answers given* on how the Hero feels after self-wetting.**

| **Session reference** | **CB 1** | | | **CB 2** | | | **CB 3** | | | **CB 4** | | | **CB 5** | | | **CB 6** | | | **CB 7** | | | **CB 8** | | | **Totals** | | | |
| --- | --- | --- | --- | --- | --- | --- | --- | --- | --- | --- | --- | --- | --- | --- | --- | --- | --- | --- | --- | --- | --- | --- | --- | --- | --- | --- | --- | --- |
| **Location**** | **P^** | **B** | **S** | **P^** | **B** | **S** | **P^** | **B** | **S** | **P^** | **B** | **S** | **P^** | **B** | **S** | **P** | **B** | **S** | **P** | **B** | **S** | **P** | **B** | **S** | **P** | **B** | **S** | **All** |
| Unhappy / won't feel good / feel bad / sad / upset / crying |  | F | C |  | C | C |  | C | C |  | C | C |  | F | C | C | C | C | C | C |  | C | C | C | 3 | 8 | 7 | 18  C:16  F:2 |
| Ashamed |  | C |  |  |  |  |  | C | C |  |  |  |  |  | C | C | F | C |  |  | C | C | C | C | 2 | 4 | 5 | 11  C:10  F:1 |
| Restless / tense |  |  |  |  |  |  |  |  |  |  | C |  |  | C |  |  | C | C | C | C | C | C | C | C | 2 | 5 | 3 | 10  C:10  F:0 |
| Afraid / scared |  |  |  |  | F | F |  | F |  |  | F |  |  |  | F |  |  |  |  | F |  |  |  | C | 0 | 4 | 3 | 7  C:1  F:6 |
| Annoyed / angry |  |  | C |  |  | C |  |  |  |  | C |  |  | C |  |  | C |  |  | C | C |  |  |  | 0 | 4 | 3 | 7  C:6  F:1 |
| Other (hot, thinking, good, happy) |  | C |  |  |  |  |  |  |  |  | C |  |  | C |  |  |  |  | C |  |  |  | C |  | 1 | 4 | 0 | 5  C:5  F:0 |
| Discomfort / Uncomfortable / Feel pain |  | C |  |  | C |  |  |  |  |  |  |  |  | F |  |  |  |  |  |  |  |  |  |  | 0 | 3 | 0 | 3  C:2  F:1 |
| Embarrassed |  |  |  |  | F | C |  |  |  |  |  |  |  |  |  |  |  |  |  |  |  |  |  | C | 0 | 1 | 2 | 3  C:2  F:1 |
| Hurt (by being laughed at / teased) |  |  | C |  |  |  |  |  | C |  |  |  |  |  |  |  |  |  |  |  |  |  |  | C | 0 | 0 | 3 | 3  C:3  F:0 |
| Troubled / worried |  |  |  |  |  |  |  |  |  |  | C | C |  |  |  | C |  |  |  |  |  |  |  |  | 1 | 1 | 1 | 3  C:3  F:0 |
| Shy |  | F |  |  | C |  |  |  |  |  |  |  |  |  |  |  |  |  |  |  |  |  |  |  | 0 | 2 | 0 | 2  C:1  F:1 |
| **Totals** |  |  |  |  |  |  |  |  |  |  |  |  |  |  |  |  |  |  |  |  |  |  |  |  |  |  |  | **72**  **C:59**  **F:13** |

*Answers given in sessions. ‘C’ indicates answer first provided by a child. ‘F’ indicates answer first provided by a facilitator.

**Location: How the hero feels after self-wetting at play (‘P’: Activity 2); home in bed (‘B’: part 1 of Activity 3); and at school (‘S’: part 2 of Activity 3).

^Session did not complete the activity.

**Table C. Answers given* on reactions of others to the Hero after self-wetting.**

| **Session reference** | **CB 1** | | **CB 2** | | **CB 3** | | **CB 4** | | **CB 5** | | **CB 6** | | **CB 7** | | **CB 8** | | **Totals** | | |
| --- | --- | --- | --- | --- | --- | --- | --- | --- | --- | --- | --- | --- | --- | --- | --- | --- | --- | --- | --- |
| **Location**** | **B** | **S** | **B** | **S** | **B** | **S** | **B** | **S** | **B** | **S** | **B** | **S** | **B** | **S** | **B** | **S** | **B** | **S** | **Total** |
| Unhappy / won't feel good / feel bad / sad / upset / crying | F |  | C | C | C | C | C | C | C |  | C | C | C |  | C |  | 8 | 4 | 12  C:11  F:1 |
| Annoyed / angry | F |  | C | C | F | F | C | C | C |  | C |  | C | C |  |  | 7 | 4 | 11  C:8  F:3 |
| Beat |  |  | C |  | C |  |  |  |  |  | C |  | C | C | C | C | 5 | 2 | 7  C:7  F:0 |
| Restless / tense |  |  |  |  | F |  | C | C |  |  | C |  | C |  | C |  | 5 | 1 | 6  C:5  F:1 |
| Scold / insult / tease |  |  |  |  | C |  |  |  | C | C |  |  | C | C |  | C | 3 | 3 | 6  C:6 |
| Troubled / worried / disturbed |  |  | F |  | C |  | C | C | C | C |  |  |  |  |  |  | 4 | 2 | 6  C:5  F:1 |
| Have to wash clothes / pati (mattress) | C |  |  |  |  |  |  |  | C |  | C |  |  |  | C |  | 4 | 0 | 4  C:4 |
| Other (thinking / happy / asked to bring Father / sent home) |  |  | C |  |  |  |  |  |  |  | F |  |  | C |  | C | 2 | 2 | 4  C:3  F:1  C:3  F:0 |
| Ashamed |  |  |  | C |  | C |  |  |  |  |  |  |  |  |  | C | 0 | 3 | 3  C:3  F:0 |
| Discomfort / Uncomfortable | C |  |  |  |  |  |  |  |  |  |  |  |  |  |  |  | 1 | 0 | 1  C:1  F:0 |
| Embarrassed |  |  |  |  |  |  |  | C |  |  |  |  |  |  |  |  | 0 | 1 | 1  C:1  F:0 |
| Hurt / feel pain |  |  | C |  |  |  |  |  |  |  |  |  |  |  |  |  | 1 | 0 | 1  C:1  F:0 |
| **Totals** |  |  |  |  |  |  |  |  |  |  |  |  |  |  |  |  |  |  | **62**  **C:55**  **F:7** |

*Answers given in sessions. ‘C’ indicates answer first provided by a child. ‘F’ indicates answer first provided by a facilitator.

**Location: Reactions to the hero self-wetting at home in bed (‘B’: part 1 of Activity 3); and at school (‘S’: part 2 of Activity 3).

**Table D. Answers given* when asked why the Hero wet the bed.**

| **Session reference** | **CB 1** | **CB 2** | **CB 3^** | **CB 4^** | **CB 5** | **CB 6** | **CB 7** | **CB 8** | **Total** |
| --- | --- | --- | --- | --- | --- | --- | --- | --- | --- |
| Drank too much water | C |  |  |  | C | C | C | C | 5  C:5  F:0 |
| Dreaming | F | C |  |  | C | C |  | C | 5  C:4  F:1 |
| Couldn't control / didn’t know | C |  |  |  | C | C |  | C | 4  C:4  F:0 |
| Couldn't reach the toilet (too far) |  |  |  |  | C | C | C | C | 4  C:4  F:0 |
| Couldn't wake-up | C |  |  |  |  |  | C | C | 3  C:3  F:0 |
| Didn't go before bed |  |  |  |  |  | C |  | C | 3  C:2  F:0 |
| No light on way to / in toilet |  |  |  |  |  |  | C | C | 2  C:2  F:0 |
| Scared to go outside | F |  |  |  |  | C |  |  | 2  C:1  F:1 |

*Answers given in sessions. ‘C’ indicates answer first provided by a child. ‘F’ indicates answer first provided by a facilitator.

^Session did not complete the activity.

**Table E. Answers given* when asked how the Hero could stop wetting the bed.**

| **Session reference** | **CB 1** | **CB 2** | **CB 3** | **CB 4** | **CB 5** | **CB 6** | **CB 7** | **CB 8** | **Total** |
| --- | --- | --- | --- | --- | --- | --- | --- | --- | --- |
| Drink less water | C | C | C | C | C | C |  | C | 7  C:7  F:0 |
| Use the toilet before bed | C | C | F | C | C | C | C |  | 7  C:6  F:1 |
| Build a latrine outside the house / closer to house |  |  |  |  | C | F | C | C | 4  C:3  F:1 |
| Call mother to be taken to the latrine | C |  | F | C |  |  |  |  | 3  C:2  F:1 |
| Go with a lamp / Have a light in toilet | C |  |  |  |  | C |  | C | 3  C:3  F:0 |
| Other (Beaten / eat less rice / toilet after waking) |  |  |  | C |  | C | C |  | 3  C:3  F:0 |

*Answers given in sessions. ‘C’ indicates answer first provided by a child. ‘F’ indicates answer first provided by a facilitator.
