# Supplementary material for: Understanding children’s experiences of self-wetting in humanitarian contexts: An evaluation of the Story Book methodology
Source: PLOS Glob Public Health. 2023 May 15;3(5):e0001194. doi: 10.1371/journal.pgph.0001194 (PMC10184904; doi:10.1371/journal.pgph.0001194)
Supplement: S4 File — Table A. Changes proposed to the Story Book session agenda. (DOCX) [file pgph.0001194.s004.docx]

# S4 File: Recommendations

## Recommendation 1: Reduce the number of activities

In Adjumani District some of the Story Book sessions ran over the intended 90 minutes (excluding play breaks) even though the suggested verbal discussions did not take place. In Cox’s Bazar all but two (CB3 and CB4) of the sessions took longer than 90 minutes, and CB3 and CB4 may only have been shorter because they did not discuss why the hero may have wet themselves. When designing the Story Book session the RT felt that 90 minutes was the maximum time that children aged five to eleven should be asked to participate, and indeed there were signs in both contexts that the children became tired and lost concentration at times. This suggests that the agenda needs to be modified to reduce the time needed.

Facilitators in Adjumani District provided a workbook to order and focus the activities (by providing drawing prompts on each page), but without such a workbook there was confusion in Cox’s Bazar about the purpose of Activity 2. Activity 1 (co-create a hero; draw the hero playing) was completed without issue, but most facilitators then moved on to explain that the hero had woken-up to find that they had wet themselves and asked the children to draw how the hero was feeling. This was the intended focus of Activity 3, and so for some groups this became Activity 3, whereas for others (CB3 and CB4 for example) the questions were repeated when they moved to Activity 3. The intention of the separate activities was to explore if the hero reacts differently after wetting themselves at different times (when playing, during the night, and at school); and to explore the different reactions of family and community members to the self-wetting (being friends when playing, caregivers at home, and teachers at school). In practice, subtleties in verbal responses in Cox’s Bazar were not detected across the different scenarios and the reactions of friends were often included when exploring self-wetting at school.

Given the confusion found regarding Activity 2 and as the sessions tended to run longer than the intended 90 minutes, it is therefore recommended that the agenda be adjusted (S2 Table). It is also suggested that groups use a workbook to guide the children through the activities (also see Recommendation 2). Verbal discussion options have been modified as per suggestions in Adjumani District that they may be better placed at the end of the session once the participants feel more comfortable, and to encourage the generation of ideas to improve humanitarian programmes.

**Table A. Changes proposed to the Story Book session agenda.**

| **Agenda item** | **Detail** |
| --- | --- |
| Activity 1: Co-creating a hero | The facilitator supports the group to create a main character, or ‘hero’, for their Story Book.  The children are asked to choose a gender, age, name, who the hero lives with, the hero’s favourite animal etc.  The facilitator draws the hero as guided by the children. |
| Activity 2: Introducing the idea of self-wetting | The children are asked to draw the hero doing an activity that makes them feel happy, for example playing football.  The facilitator then explains that after playing the hero goes home to bed, but wakes to find that they have wet themselves. The children are asked to draw what the hero does now.  The children are then asked to draw what the hero’s caregiver does when they find that the hero has wet the bed.  *Verbal discussion options:* Reasons why the hero wet themselves (and ideas for improving the day of the hero if the facilitator chooses not to do Activity 3). |
| Activity 3: Exploring a further self-wetting episode | *The facilitator may choose not to do this activity depending on the time taken to complete Activities 1 and 2.*  The facilitator explains that the hero has now gone to school and wets themselves there. The children are asked to draw what the hero now does.  The children are then asked to draw what the hero’s teacher and friends do when they find out that the hero has wet themselves.  *Verbal discussion options:* Reasons why the hero wet themselves (if not already discussed) and ideas for improving the day of the hero |

## Recommendation 2: Simplify the drawing activities

The children (and at times, the facilitators), struggled to draw the emotions of the hero and the caregiver and/or teacher. The activities have been updated to ask the children to draw activities rather than emotions (S2 Table). It is also recommended that a workbook is provided for each child, with a page per activity that includes drawing prompts. This could be outlines, as successfully used in Adjumani District, and/or a series of culturally appropriate emojis which the children can indicate as being appropriate (for example, by circling). These could also be referred to if needed to support any verbal discussions. The emojis provided should include a range of activities and emotions so that they do not guide the children to a specific answer, but not so many that the children are overwhelmed. Using such prompts may also support interpretation of the drawings by researchers to ensure that subsequent programming recommendations are appropriate.

## Recommendation 3: Use the Story Book sessions to normalise self-wetting

There may be an opportunity to better use the sessions to educate children on key messages such as how prevalent self-wetting is, and the fact that most children grow out of the condition. Such information could help to reassure any children experiencing self-wetting and also help to lessen any stigma associated with self-wetting. As suggested in Adjumani District these could even be incorporated into songs or stories to be used as ice-breakers. Similarly, at the end of the session the facilitator could provide the name of someone as a first contact to getting support for self-wetting in the home. The children could be advised that they can approach this person themselves, tell the name to their caregiver, and/or tell anyone they know that may need such support.
