# Supplementary material for: Understanding children’s experiences of self-wetting in humanitarian contexts: An evaluation of the Story Book methodology
Source: PLOS Glob Public Health. 2023 May 15;3(5):e0001194. doi: 10.1371/journal.pgph.0001194 (PMC10184904; doi:10.1371/journal.pgph.0001194)
Supplement: Attachment — Submitted filename: Response to reviewers.docx [file pgph.0001194.s005.docx]

**‘Understanding children’s experiences of self-wetting in humanitarian contexts: An evaluation of the Story Book methodology’: Responses to review comments**

| **Correction** | **Changes made** | **Page** |
| --- | --- | --- |
| **Journal requirements** | | |
| 1. Please include a complete copy of PLOS’ questionnaire on inclusivity in global research in your revised manuscript. Our policy for research in this area aims to improve transparency in the reporting of research performed outside of researchers’ own country or community. The policy applies to researchers who have travelled to a different country to conduct research, research with Indigenous populations or their lands, and research on cultural artefacts. The questionnaire can also be requested at the journal’s discretion for any other submissions, even if these conditions are not met.  Please find more information on the policy and a link to download a blank copy of the questionnaire here: [https://journals.plos.org/globalpublichealth/s/best-practices-in-research-reporting](https://eur03.safelinks.protection.outlook.com/?url=https%3A%2F%2Fjournals.plos.org%2Fglobalpublichealth%2Fs%2Fbest-practices-in-research-reporting&data=05%7C01%7Ccncr%40leeds.ac.uk%7C70bc8b930ec14072d27708dacb54d349%7Cbdeaeda8c81d45ce863e5232a535b7cb%7C1%7C0%7C638045862445658249%7CUnknown%7CTWFpbGZsb3d8eyJWIjoiMC4wLjAwMDAiLCJQIjoiV2luMzIiLCJBTiI6Ik1haWwiLCJXVCI6Mn0%3D%7C3000%7C%7C%7C&sdata=emB%2BNU3GVBBEqBQTb1Z5ntxDOZ8KjHK3%2BvRXwNynczc%3D&reserved=0). Please upload a completed version of your questionnaire as Supporting Information when you resubmit your manuscript. | A questionnaire has been completed and uploaded as Supporting Information, and reference to this has been included in the main body of the Manuscript. | 16 |
| 2. Please send a completed 'Competing Interests' statement, including any COIs declared by your co-authors. If you have no competing interests to declare, please state "The authors have declared that no competing interests exist". Otherwise please declare all competing interests beginning with the statement "I have read the journal's policy and the authors of this manuscript have the following competing interests:" | The following ‘Competing Interests’ statement has been submitted:  The authors declare only the following which may be considered as a potential non- financial competing interest: Mahbub-Ul Alam is currently serving on the editorial board of PLOS Global Public Health. | n/a |
| 3. Please amend your detailed Financial Disclosure statement. This is published with the article. It must therefore be completed in full sentences and contain the exact wording you wish to be published.  a. State the initials, alongside each funding source, of each author to receive each grant. b. If any authors received a salary from any of your funders, please state which authors and which funders.  If you did not receive any funding for this study, please simply state: “The authors received no specific funding for this work.” | The Financial Disclosure Statement has been updated. | n/a |
| 4. Please provide separate figure files in .tif or .eps format only and remove any figures embedded in your manuscript file. Please also ensure that all files are under our size limit of 10MB.  For more information about figure files please see our guidelines:  [https://journals.plos.org/globalpublichealth/s/figures](https://eur03.safelinks.protection.outlook.com/?url=https%3A%2F%2Fjournals.plos.org%2Fglobalpublichealth%2Fs%2Ffigures&data=05%7C01%7Ccncr%40leeds.ac.uk%7C70bc8b930ec14072d27708dacb54d349%7Cbdeaeda8c81d45ce863e5232a535b7cb%7C1%7C0%7C638045862445658249%7CUnknown%7CTWFpbGZsb3d8eyJWIjoiMC4wLjAwMDAiLCJQIjoiV2luMzIiLCJBTiI6Ik1haWwiLCJXVCI6Mn0%3D%7C3000%7C%7C%7C&sdata=iMS5QI3lwn9i6%2BIz4EPmC2Ypb0uZuYzdHHRYCGT9Alc%3D&reserved=0)  [https://journals.plos.org/globalpublichealth/s/figures#loc-file-requirements](https://eur03.safelinks.protection.outlook.com/?url=https%3A%2F%2Fjournals.plos.org%2Fglobalpublichealth%2Fs%2Ffigures%23loc-file-requirements&data=05%7C01%7Ccncr%40leeds.ac.uk%7C70bc8b930ec14072d27708dacb54d349%7Cbdeaeda8c81d45ce863e5232a535b7cb%7C1%7C0%7C638045862445658249%7CUnknown%7CTWFpbGZsb3d8eyJWIjoiMC4wLjAwMDAiLCJQIjoiV2luMzIiLCJBTiI6Ik1haWwiLCJXVCI6Mn0%3D%7C3000%7C%7C%7C&sdata=Qu7wDd4xfD%2BVg9nIabEev%2BQOwQ0hnhyJPncRPZxs1rY%3D&reserved=0) | Figure files have been removed from the Manuscript. |  |
| While revising your submission, please upload your figure files to the Preflight Analysis and Conversion Engine (PACE) digital diagnostic tool, [https://pacev2.apexcovantage.com/](https://eur03.safelinks.protection.outlook.com/?url=https%3A%2F%2Fpacev2.apexcovantage.com%2F&data=05%7C01%7Ccncr%40leeds.ac.uk%7C70bc8b930ec14072d27708dacb54d349%7Cbdeaeda8c81d45ce863e5232a535b7cb%7C1%7C0%7C638045862445658249%7CUnknown%7CTWFpbGZsb3d8eyJWIjoiMC4wLjAwMDAiLCJQIjoiV2luMzIiLCJBTiI6Ik1haWwiLCJXVCI6Mn0%3D%7C3000%7C%7C%7C&sdata=bNE7DSrkKczwgNLaGHAYXk%2BBKIC1mfZsp8er5utdN94%3D&reserved=0). PACE helps ensure that figures meet PLOS requirements. To use PACE, you must first register as a user. Registration is free. Then, login and navigate to the UPLOAD tab, where you will find detailed instructions on how to use the tool. If you encounter any issues or have any questions when using PACE, please email PLOS at [figures@plos.org](mailto:figures@plos.org). Please note that Supporting Information files do not need this step. | This has been done and new files have been uploaded. | n/a |
| **Reviewer #1** |  |  |
| Even if it emerges across the paper, I recommend to state more clearly why self-wetting was chosen as a problem to be investigated: consequences are described, but what are its dimensions, occurrence, why it is worthy to be investigated in that context? | The Background section has been expanded to include why self-wetting is worthy of investigation in this context. | 4 |
| Also it will be beneficial to describe more why the Story Book was chosen to be tested as methodology and not another methodology. Due to the fact that the Story Book is not the best methodology to address this problem in humanitarian settings, which methods can be appropriate? or what about next steps to identify more appropriate tools for such contexts? | A detailed description of the development of the Story Book methodology can be found in S1. This has been noted in the Manuscript. Further detail has also been added to Table 1.  Recommendations have been expanded in S3 to develop the Story Book methodology so that it’s a more appropriate tool for this context. | 8  S3 |
| **Reviewer #2** |  |  |
| The paper purpose is highly important and is recommended to be further improved especially for sensitive topics.  However, because this is a methodological paper, it is important that the methodology part of the paper needs to be more detailed in order for the recommendations and conclusion made in the paper to be justified and understandable .  1. The setting; (place) in which the sessions were conducted with their children. Was it in their homes, schools, or unfamiliar places, were the children in each session familiar with each other before the sessions, were the parents( or other caregivers) of the children around during the sessions etc. All these factors may affect children's response during the sessions.  2. The methodology needs to include the description of the facilitators of the sessions. How were they selected? This should include their gender, educational level, experience in research with children, their previous level of interaction or familiarity with the children and the community.  3. Were local languages used with the children  4. What is the socioeconomic status or educational level of the children involved in each session, Do children in each session have similar levels of socioeconomic or educational status.  5 What is the total number of children participants. Descriptive statistics ( gender, age) of the participant should be included.  6. Asides the table 2 that shows that sessions were done in age groups. It seems that two age groups were used from the table ; the text should also indicate how children were grouped for the sessions.  7. Do the children differ in participation level during the sessions based on their age groups. Please include this in the result sessions | The Methods section has been expanded to address 1-5.  Observations regarding 7. can be found in the Results section under ‘Acceptability’. | 12  19 |
